# Generation of Quantum Vortex Electrons with Intense Laser Pulses

**DOI:** 10.1002/advs.202404564

**Published:** 2024-09-03

**Authors:** Zhigang Bu, Liangliang Ji, Xuesong Geng, Shiyu Liu, Shaohu Lei, Baifei Shen, Ruxin Li, Zhizhan Xu

**Affiliations:** ^1^ State Key Laboratory of High Field Laser Physics Shanghai Institute of Optics and Fine Mechanics (SIOM) Chinese Academy of Sciences (CAS) Shanghai 201800 China; ^2^ University of Chinese Academy of Sciences Beijing 100049 China; ^3^ Department of Physics Shanghai Normal University Shanghai 200234 China; ^4^ Shanghai Tech University Shanghai 201210 China

**Keywords:** intense laser pulse, orbital angular momentum, quantum electrodynamics, quantum vortex state, radiation reaction

## Abstract

Accelerating a free electron to high‐energy forms the basis for studying particle and nuclear physics. Here it is shown that the wave function of such an energetic electron can be further manipulated with the femtosecond intense lasers. During the scattering between a high‐energy electron and a circularly polarized laser pulse, a regime is found where the enormous spin angular momenta of laser photons can be efficiently transferred to the electron orbital angular momentum (OAM). The wave function of the scattered electron is twisted from its initial plane‐wave state to the quantum vortex state. Nonlinear quantum electrodynamics (QED) theory suggests that the GeV‐level electrons acquire average intrinsic OAM beyond ⟨l⟩∼100ℏ at laser intensities of 10^20^ W cm^−2^ with linear scaling. These electrons emit *γ*‐photons with two‐peak spectrum, which sets them apart from the ordinary electrons. The findings demonstrate a proficient method for generating relativistic leptons with the vortex wave functions based on existing laser technology, thereby fostering a novel source for particle and nuclear physics.

## Graphical abstract

Wave functions of high‐energy electrons can be manipulated with ultra‐short intense laser pulses. Nonlinear scattering theory illustrates the efficient transfer of numerous spin angular momenta of laser photons to orbital angular momenta of scattered electrons, twisting electron wave functions into quantum vortex states. This mechanism fosters a novel source for particle and nuclear physics.

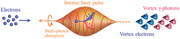

## 1 Introduction

High‐energy particles, such as relativistic electrons, play a crucial role in advancing our understanding of the subatomic world. These particles undergo acceleration to attain substantial momentum in free space and then collide with nuclei or other free particles. In many instances, their behavior is effectively described using the plane‐wave states, which are eigenstates of the energy‐momentum and the spin. Both features have been extensively utilized in the fields of particle and nuclear physics. In recent years, it has been found that the wave functions of not only photons but also charged particles can be reshaped, by the spiral phase plates,^[^ ^1^, ^2^, ^3^, ^4^ ^]^ fork gratings,^[^ ^3^, ^5^ ^]^ or free electron lasing,^[^ ^6^, ^7^, ^8^, ^9^ ^]^ to form coherent vortex states carrying intrinsic orbital angular momenta (OAM). Electrons with vortex wave functions (referred to as the quantum vortex states, QVS) and kinetic energies of hundreds of keV have been realized in transmission electron microscopes,^[^ ^10^, ^11^, ^12^, ^13^ ^]^ enabling enhanced resolution in exploring magnetic crystals and chiral materials.^[^ ^14^, ^15^ ^]^ Yet these approaches are only limited to the non‐relativistic electrons with energies much smaller than MeV. It remains unclear whether the wave functions of highly relativistic particles can be intentionally manipulated within existing accelerators or colliders, since their wavelengths are too small for any structures accessible with current technologies.

Here we show that the intense laser pulses are efficient in producing relativistic electrons carrying large intrinsic OAM. It relies on the scattering of electrons with multiple laser photons in a circularly polarized (CP) pulse, i.e., the nonlinear Inverse Compton Scattering (NCS). The absorption of numerous laser photons by the electron not only leads to the transfer of momentum to the final particles but also angular momentum (AM). It has been shown in NCS that the emitted γ‐photon could inherit the intrinsic OAM from the AM of laser photons while its energy is blueshifted.^[^ ^16^, ^17^, ^18^ ^]^ However, the high‐energy electrons involved in the interaction are considered unable to gain net intrinsic OAM.^[^ ^17^, ^18^, ^19^, ^20^, ^21^, ^22^ ^]^

We find a new regime where the electron gains a significant portion of AM during NCS and carries intrinsic OAM. Our vortex scattering theory reveals that through radiation‐reaction (RR) effect in the strongly nonlinear regime, the average intrinsic OAM obtained by GeV‐level electrons is proportional to the laser intensity, achieving beyond 100ℏ at a laser intensity of 10^20^ W cm^−2^. With these lasers readily accessible in many facilities worldwide, the mechanism provides a new approach to manipulate the wave functions of highly relativistic electrons or positrons. It opens up new possibilities for investigating AM physics in nuclear systems^[^ ^23^, ^24^ ^]^ and creating exotic high‐AM particle states in colliders relevant to high‐energy physics, which are not possible with the ordinary sources.

## 2 Results and Discussion

### 2.1 Generating Vortex‐Featured Electron from Non‐Vortex Electron

**Figure 1:**
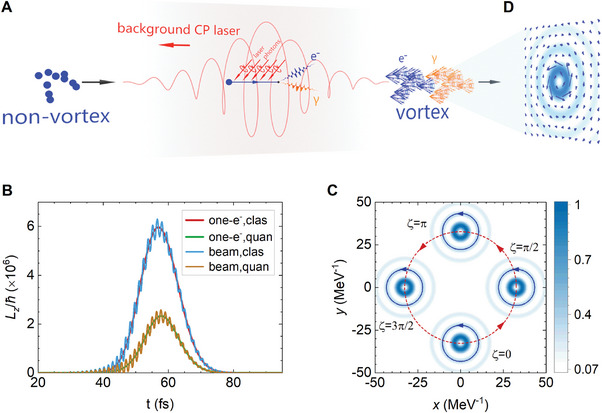
Vortex NCS process and OAM features of QVS electrons dressed in a CP laser pulse. A) Schematic diagram of the vortex NCS in the CP laser fields. A high‐energy electron absorbs *N* laser photons and emits a QVS γ‐photon during the scattering, it gains net intrinsic OAM and its wave‐function is twisted into vortex state. B) Time evolution of one‐electron and electron beam OAMs when moving in a CP laser pulse with classical radiation and quantum radiation. C) Time evolution of the transverse probability density of Volkov‐Bessel state in one laser period, red circle denotes the extrinsic circular motion driven by the CP laser field, blue circle denotes the intrinsic OAM of the vortex state. D) Transverse current density of QVS electron in CP laser field. The laser amplitude and helicity are *a*_0_ = 1 and *λ*_0_ = 1. Electron OAM and spin numbers are *l* = 1 and *s* = 1, its energy (relativistic factor) is *γ_p_* = 10^4^ and the opening angle is defined by transverse and longitudinal momenta: tan*θ_p_* = *p*_⊥_/*p_z_*.

The concept is depicted in **Figure** **1**A, where a GeV‐level electron beam collides with a CP laser pulse, the vortex‐featured electrons and γ‐photons are generated after the scattering. In a classical or semi‐classical picture, a point‐like electron in the CP laser undergoes a helical motion and acquires incoherent and extrinsic OAM.^[^ ^25^, ^26^, ^27^, ^28^ ^]^ However, this motion cannot be preserved after the interaction. As an example, we show the time evolution of OAMs from a single electron or electron beam in the CP‐laser‐electron interaction (Figure 1B). Here the electron radiation and induced reaction effect are considered with the classical Landau–Lifshitz model^[^ ^29^ ^]^ and the stochastic radiation model,^[^ ^30^, ^31^, ^32^ ^]^ respectively. In these cases, the OAM value is derived by counting ***r*** × ***p*** for each point‐like particle, hence dependent on the choice of axis and referred to as extrinsic.^[^ ^33^, ^34^ ^]^ We see that OAMs in all cases increase in the first half of the laser pulse and then decline in the second. Eventually, the electrons do not acquire net extrinsic OAM as point‐like particles after the interaction. It is also impossible to learn any information about the intrinsic properties of particle states in classical and semi‐classical theory.

To find this out, we move to the quantum realm by describing the particles with wave functions. In contrast to the widely used plane‐wave state, the wave function here must include the quantum numbers relevant to OAM, which can be characterized by the vortex state in Bessel mode.^[^ ^35^ ^]^ Further, the state is dressed in an intense laser field during the interaction — often described by the Volkov state.^[^ ^36^ ^]^ Combining both features, we construct the electron QVS dressed in a CP laser field, $\psi_{p_{⊥},p_{z}}^{s,l}\left(x\right)$, by coherent superposition of the Volkov states $\psi_{p}^{s}\left(x\right)$, through helical Fourier spectrum (see in Experimental Section). This wave function, known as Volkov‐Bessel state, is depicted by electron energy and momenta (*E_p_*,*p*_⊥_,*p_z_*), intrinsic OAM number *l* and SAM number *s*. It is not an eigenstate of any AM projection due to the external field. Yet one can calculate the mean value of the OAM projection onto the propagation z‐axis, 〈*L_z_* 〉 =  *l* + Δ*l*. Here the quantum number *l* represents the intrinsic OAM number, and Δ*l* is determined by the laser field strength and the electron momentum, i.e., it is associated with the extrinsic OAM and spin‐orbit interaction (SOI) dressed by the laser field. The differences can be seen clearly from the probability density ρ^*s*,*l*^(*x*) of the electron state and its evolution in one phase period [0,2π] (Figure 1C). At each phase, the concentric ring profile is well retained, corresponding to the intrinsic vortex structure of the electron state. Meanwhile, the entire density distribution rotates around a central axis for a cycle within one phase period. It is a typical motion of an electron moving in the CP laser field, generating the extrinsic OAM Δ*l*.

Using the QVS, we can see how a vortex‐featured electron is generated from a non‐vortex one. The transverse current density of the QVS electron can be divided into the intrinsic and extrinsic components, $j_{⊥}^{s,l}\;\left(x\right)=j_{⊥,int}^{s,l}\;\left(x\right)+j_{⊥,ext}^{s,l}\left(x\right)$. The intrinsic current vector shows a distinctive vortex structure (Figure 1D). When an electron leaves the laser field after the scattering, the extrinsically rotational motion (extrinsic current density) vanishes, while the intrinsic vortex structure is retained and simplified to the SOI of a free QVS electron.^[^ ^35^ ^]^ In other words, the preservation of OAM manifests as the electron state becoming twisted into QVS.

**Figure 2:**
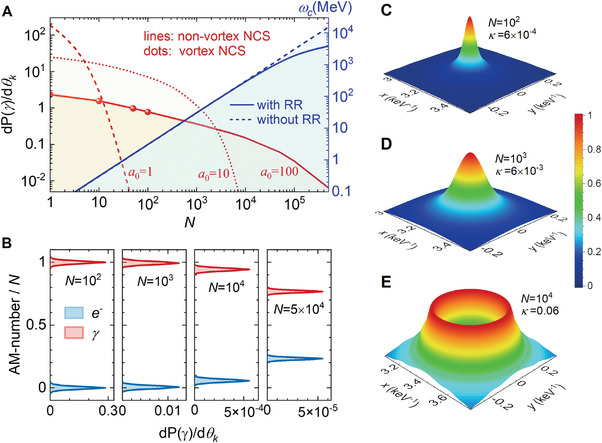
Generating vortex‐featured electrons from non‐vortex electrons in vortex NCS. A) Differential emission rate at the opening angle *θ*_0_ = *a*_0_/*γ_Q_* versus *N* calculated by the vortex scattering theory (red dots, summing over all AM channels) and non‐vortex theory (red lines), and they give equal results. The red dashed, dotted, and solid lines are the emission rates with the laser amplitudes of *a*_0_ = 1, 10, and 100. The helicity of the laser field is *λ*_0_ = 1 and the incident electron energy is *γ_Q_* = 10^4^. The blue lines represent the central energy of γ‐photons. B) Intrinsic AM distribution of the scattered electrons and γ‐photons when absorbing *N* = 10^2^, 10^3^, 10^4^, and 5 × 10^4^ laser photons. It is obvious that when *N* > 10^4^, the AMs of electrons and γ‐photons start to show a noticeable redistribution. C–E) Normalized transverse density of the scattered electrons for *N* = 10^2^, 10^3,^ and 10^4^. They undergo a transition from a Gaussian‐like distribution to a hollow structure as *N* increases.

The twist of the electron wave function is quantitatively analyzed in the framework of quantum electrodynamics (QED), where the scattering probabilities are obtained based on the Volkov‐Bessel state. The multi‐photon NCS is then ʻsimplifiedʼ to the decay of a non‐vortex electron into a QVS photon and a QVS electron in the CP laser field (NV→V+V process). Let us consider the laser pulse with frequency ω_0_ =  1.55 eV, helicity λ_0_ = ±1, intensity *I*_0_ = 2.14 × 10^22^ W cm^−2^, normalized amplitude *a*_0_ = |*e*|*A*_0_/*M* = 100 (*e* and *M* are the charge and mass of electron, *A*_0_ is the vector potential amplitude of the laser field) and duration *L* = 26 fs propagating along the z‐axis and colliding head‐on with a relativistic electron. The initial electron state is described as a wavepacket form of the Volkov state, $\Psi_{E_{Q},Q_{z}}^{\sigma }\;\left(x\right)=\frac{1}{{\left(2\pi \right)}^{3/2}}\;\int d^{3}q\rho \left(q_{⊥},q_{z}\right)\psi_{q}^{\sigma }\left(x\right)$, where $\rho \left(q_{⊥},q_{z}\right)∼exp\left[-4q_{⊥}^{2}/\tau_{⊥}^{2}-4{\left(q_{z}-Q_{z}\right)}^{2}/\tau_{z}^{2}\right]$ is a Gaussian weighting function, *Q_z_* is the central longitudinal momentum, and *E_Q_* = 5.11 GeV is the central energy (relativistic factor γ_*Q*_ = 10^4^ ), *τ*_⟂_ and *τ_z_* are the transverse and longitudinal widths of the wave packet, *σ* describes the electron spin. The opening angle of emitted QVS γ‐photon defined by tan θ_*k*_ = *k*_⊥_/|*k_z_* | is around θ_*k*_ = θ_0_  = *a*_0_/γ_*Q*_  for γ_*Q*_ ≫ *a*_0_.^[^ ^37^ ^]^ The AM‐dependent differential scattering rate is derived from the S‐matrix of the scattering, $\mathrm{dP}=|S_{\mathrm{fi}}^{V}{|}^{2}\;p_{⊥}k_{⊥}dp_{⊥}dp_{z}dk_{⊥}dk_{z}$. When absorbing *N* laser photons, the differential emission rate at the opening angle *θ*_0_ can be obtained by integrating the momenta of the scattered electrons and summing over all possible AM channels (**Figure** **2**A). Smaller laser intensity causes the emission rate to decay more rapidly with *N* and the average number of absorbed photons is lower in this case due to the suppression of nonlinear effects. We compared the γ‐photon emission rate calculated under the vortex (red dots) and non‐vortex (red line) theories, and find that switching the wave functions of final particles from the non‐vortex states to the QVS does not change the total emission rate. This indicates that the vortex scattering theory is complete. When absorbing *N* laser photons, the central energy of emitted γ‐photon at *θ_k_* angle can be approximated obtained by using the energy and momentum conservation condition,

(1) $$\begin{array}{c} \omega_{c}\approx \frac{N\omega_{0}\left(E_{Q}-Q_{z}\right)}{\left({\tilde{E}}_{Q}+N\omega_{0}\right)+\left({\tilde{Q}}_{z}+N\omega_{0}\right)\cos\theta_{k}} \end{array}$$

as illustrated by the blue line in Figure 2A, where *Q_z_* is the electron central momentum along z‐axis, ${\tilde{E}}_{Q}$ and ${\tilde{Q}}_{z}$ are electron quasi‐energy and quasi‐momentum in the external CP laser field. In the limit *a*_0_ ≪ 1, θ_*k*_ → 0 and *N* → 1, it simplifies to the well‐known linear form $\omega_{c}\to 4\gamma_{Q}^{2}\omega_{0}$.

While the momenta of *N* laser photons are absorbed and converted to the emitted γ‐photon following Equation (1), the enormous SAMs are also necessarily transferred to final particles and twist their wave functions into vortex states. The connection between *N* and intrinsic OAM number, *l*, of the scattered electron and the total angular momentum (TAM) number, *j*, of the γ‐photon follows the conservation law *Nλ*_0_ = *l*+*j*+Δ, where Δ = 0, ±1 depends on whether the electron spin is flipped during the scattering. Under the parameter region considered here, the electron spin‐flip rates are negligible, thus we get *Nλ*_0_ = *l*+*j*. To reveal the vortex features of the scattering, we decompose the emission rate in Figure 2A into various AM channels under vortex theory and obtain the AM distribution (Figure 2B). The normalization by *N* clearly shows the proportion of AM taken by the final particles from the total SAM of absorbed laser photons. In the weakly nonlinear regime when *N* is small, *N* ≪ 10^4^, almost all the SAMs from the laser photons are transferred to the γ‐photon and the scattered electron does not acquire the intrinsic OAM. It agrees with the AM properties in linear Compton scattering^[^ ^16^ ^]^ where the electron efficiently transfers the TAM of the incoming photon to the emitted photon while its own state remains minimally disturbed.

The allocation shifts significantly when entering the highly nonlinear regime. When *N* increases to more than 10^4^ the SAMs of the laser photons are gradually transferred to the scattered electron, causing the central TAM number of γ‐photon distribution to shift from 1. The peak probability of electrons is at *l* = 572 when *N* = 10^4^, and its width is Δ*l* = 300. When *N* increases to 5 × 10^4^, the probability is distributed around *l* = 11650 with the width of Δ*l* = 1100. This indicates that the scattered electrons are in a mixed OAM state rather than a pure OAM state. Comparing the trend of photon central energy (Figure 2A), one notices that the electron starts to gain significant OAM when ω_*c*_ deviates from the blue dashed line where the back reaction from the γ‐photon emission is omitted (referred to as the non‐RR case). We introduce a parameter κ  = ω_*c*_/*E_Q_* , defined as the ratio of the central energy of γ‐photon to the incoming electron energy. Not only does this parameter govern the recoil effect on electron due to the emission of γ‐photon, but it also determines the central OAM number of scattered electrons through the scaling

(2) $$\begin{array}{c} \;l_{c}=\;\kappa N \end{array}$$

and the corresponding central TAM number of γ‐photon is *j_c_* =  *N*(1−κ). To see this, we rewrite Equation (1) as $\omega_{c}\approx 2N\omega_{0}\gamma_{Q}/\left(a_{0}^{2}/\gamma_{Q}+2N\omega_{0}/M\right)$. When $N\omega_{0}/M\ll a_{0}^{2}/\gamma_{Q}$, parameter κ ≪ 1 and the photon central energy increases linearly with *N*, which is the non‐RR regime. When *N* is approaching $\left(a_{0}^{2}/\gamma_{Q}\right)\left(M/2\omega_{0}\right)∼10^{5}$, we have κ∼1 and the RR effect becomes apparent. In this regime, the electron wave functions are significantly twisted and their density profiles undergo a transition from a Gaussian‐like distribution to a hollow structure (Figure 2C–E), a typical sign coinciding with the quantum vortex structure and the electron OAM distribution (Figure 2B). Therefore, the generation of vortex‐featured electrons in NCS is a novel effect triggered by the RR effect in the quantum regime. This observation signifies the first acknowledgment of quantum RR affecting not just momentum but also angular momentum.

**Figure 3:**
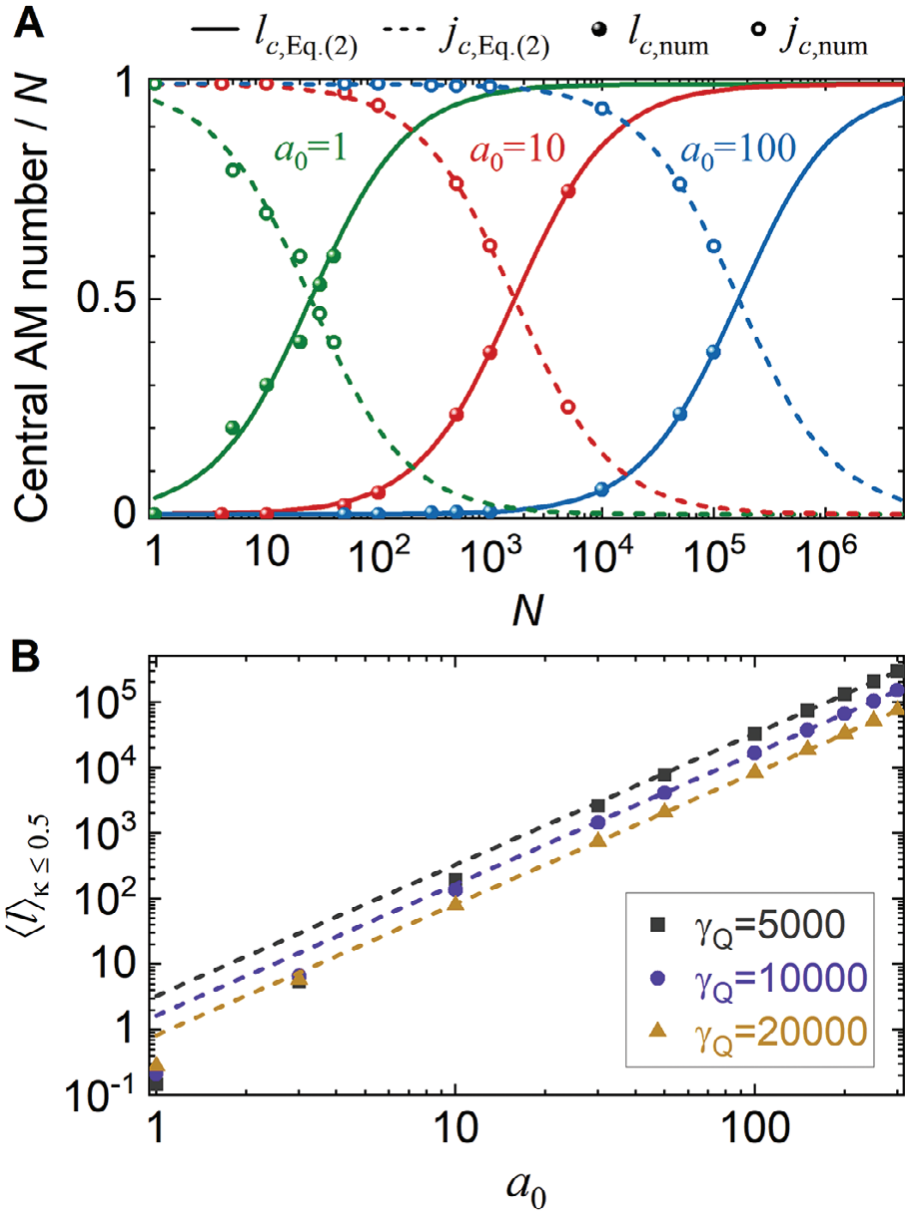
AM distribution of the scattered particles. A) Central AM numbers of the scattered electrons and γ‐photons versus the number of absorbed photons *N*. The laser amplitude is *a*_0_ = 1 (green), 10 (red), and 100 (blue), and the energy of the incident electron is *γ_Q_* = 10^4^. The lines represent the scaling law, Equation (2); dots are numerical results from S‐matrix. B) Average value of the scattered electron's intrinsic OAM calculated in the range of *κ* ≤ 0.5, 〈 ***l*** 〉_***κ*** ≤ 0.5_. The dashed lines represent the $\propto a_{0}^{2}$ scaling.

The central AM numbers of the electron and γ‐photon given by the scaling law, Equation (2), can be confirmed by numerical results calculated from S‐matrix theory, in good agreement for a large range of the laser intensities (**Figure** **3**A). Absorption of more laser photons causes an increase in the energy of emitted photon, thus *κ* gradually becomes non‐negligible and the electron wave function is twisted more significantly. A turning point appears at *κ* = 0.5 where RR is strong enough to cause the electron OAM to surpass the photon TAM. Beyond this point, the growth in photon TAM tends to saturate while the electron OAM gradually approaches |*l_c_* | → *N*.

The mechanism revealed here is rather efficient. We count the average value of intrinsic OAMs of the scattered electrons as a function of the drive laser amplitude (Figure 3B). Numerical results (dots) indicate that when laser amplitude *a*_0_ > 30, the average intrinsic OAM scales approximately as $\left\langle l\right\rangle \propto a_{0}^{2}$. At the laser intensity of 10^20^ W cm^−^^2^, one achieves an average OAM of ⟨l⟩∼100ℏ. Notably, this level of intensity is readily attainable with 100TW‐class laser systems, a capability already realized in numerous laser facilities. With state‐of‐the‐art PW‐class lasers, the average OAM approaches the order of 10^4^ ℏ. There is no discernible significant limit for the average OAM value within the considered parameter regions.

### 2.2 Scattering of QVS Electron

The scattered high‐energy electron with a vortex wave function may undergo further scattering, invoking the V→V + V process. To resolve this, we describe the incoming QVS electron as a wave packet of Volkov‐Bessel state carrying the SAM and OAM numbers of $\tilde{s}$ and $\tilde{l}$, $\Psi_{Q_{⊥},Q_{z}}^{\tilde{s},\tilde{l}}\;\left(x\right)=\int dq_{z}dq_{⊥}\sqrt{q_{⊥}}\rho \left(q_{⊥},q_{z}\right)\psi_{q_{⊥},q_{z}}^{\tilde{s},\tilde{l}}\left(x\right)$, with a Gaussian weighting function $\rho \left(q_{⊥},q_{z}\right)∼exp\left[-4{\left(q_{⊥}-Q_{⊥}\right)}^{2}/\tau_{⊥}^{2}-4{\left(q_{z}-Q_{z}\right)}^{2}/\tau_{z}^{2}\right]$ and the central momenta *Q*_⟂_ and *Q_z_*. The electron central energy and momenta satisfy $E_{Q}=\;M\gamma_{Q}=\sqrt{Q_{⊥}^{2}+Q_{z}^{2}+M^{2}}$.

**Figure 4:**
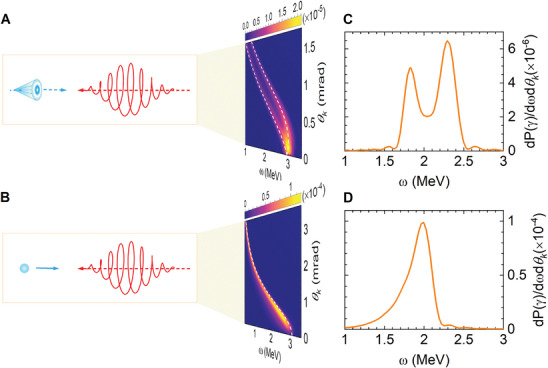
Emission spectra of QVS and non‐vortex electrons in the CP laser field. The laser amplitude and helicity are *a*_0_ = 1 and *λ*_0_ = 1. A) Emission spectrum of QVS electrons. The intrinsic OAM number of the incoming QVS electron is $\tilde{l}=\;1$, its central energy and transverse momentum are ***γ***_***Q***_ = 10^3^  and ***Q***_⊥_ =  2 × 10^−4^ ***E***_***Q***_. It shows a two‐peak distribution. B) Emission spectrum of non‐vortex electrons with the same central energies. The white dashed lines represent the theoretically central energy of γ‐photons. C,D) Energy spectra at emission opening angle *θ_k_* = 1 mrad for the incident QVS and non‐vortex electrons.

There is a significant difference in emission spectra between the vortex and non‐vortex electrons, even in a weakly nonlinear regime. Considering the laser amplitude is *a*_0_ = 1, the OAM number of incoming QVS electrons is $\tilde{l}=\;1$, its central energy and transverse momentum are γ_*Q*_ = 10^3^  and *Q*_⊥_ =  2 × 10^−4^ *E_Q_*. The energy spectrum of emitted γ‐photons shows a two‐peak distribution (**Figure** **4**A). In contrast, the NV→V+V process produces a single‐peaked emission spectrum (Figure 4B). The energy spectra at *θ_k_* = 1mrad are compared in Figure 4C,D. The two‐peak structure is attributed to the opening angles of the incident QVS electron *θ_q_* and the emitted photon *θ_k_*. They form two angle configurations, α_1_ =  |θ_*k*_ − θ_*q*_| and α_2_ = θ_*k*_  + θ_*q*_, resulting in two central energies of the emitted γ‐photons,

(3) $$\begin{array}{c} \omega_{\pm }\approx \frac{N\omega_{0}\left(E_{Q}-Q_{z}\right)}{\left({\tilde{E}}_{Q}+N\omega_{0}\right)+\left({\tilde{Q}}_{z}+N\omega_{0}\right)cos\left(\theta_{k}\mp \theta_{q}\right)} \end{array}$$

Equation (3) is depicted by the white dashed lines in Figure 4A,B, they are in good agreement with the spectrum distribution. When θ_*q*_ → 0, the incoming QVS electron degenerates into a non‐vortex electron, and Equation (3) is simplified to Equation (1). For a quasi‐monoenergetic electron beam, the two‐peak spectrum can serve as a distinct signal to identify the QVS electrons from the non‐vortex ones, which can be achieved with current experimental techniques.

## 3 Conclusion

We demonstrated a new mechanism that leads to the twist of relativistic electron wave function using a CP‐intense laser pulse. It is found that in the highly nonlinear regime, the RR effect dominates the transfer of intrinsic AM in vortex NCS. We developed the first full‐vortex scattering theory in the Furry picture of QED and found that the SAMs of the laser photons are mostly carried away by the emitted γ‐photon in the weakly nonlinear regime. However, in the laser field with higher intensity, the triggering of RR effect drives the scattered electrons to gain a higher portion of the intrinsic AMs. The average intrinsic OAM acquired by the scattered electrons scales approximately linear with the laser intensity. Compared to single‐photon processes, the intense laser pulse induces strong nonlinear effects (multiphoton absorption process), which significantly enhance the generation efficiency of the vortex‐featured electrons. The results show that when *a*_0_ > 10, more than 90% of the scattered electrons gain intrinsic OAMs. After the interaction, these OAMs are effectively preserved and the electron wave functions are twisted into QVSs. Our work provides a viable scheme for manipulating the one‐particle vortex state of the high‐energy electron with current laser technology, which paves the way for exploring intrinsic OAM and vortex effects in high‐energy particle and nuclear physics.

## 4 Experimental Section

### Electron QVS in CP Laser Field (Volkov‐Bessel State)

The electron Volkov‐Bessel state, $\psi_{p_{⊥},p_{z}}^{s,l}\left(x\right)$, dressed in a CP laser field was expressed as the superposition of Volkov states $\psi_{p}^{s}\left(x\right)$,^[^ ^38^, ^39^ ^]^

(4) $$\begin{array}{c} \psi_{p_{⊥},p_{z}}^{s,l}\;\left(x\right)=\int d{\tilde{p}}_{z}d\phi_{\tilde{p}}d{\tilde{p}}_{⊥}{\tilde{p}}_{⊥}\psi_{\tilde{p}}^{s}\left(x\right)f_{p_{⊥},p_{z}}^{l}\left(\tilde{p}\right) \end{array}$$

where $f_{p_{⊥},p_{z}}^{l}\;\left(\overset{∼}{p}\right)=\left(1/\sqrt{2\pi }i^{l}{\tilde{p}}_{⊥}\right)\;\delta \left({\tilde{p}}_{⊥}-p_{⊥}\right)\delta \left({\tilde{p}}_{z}-p_{z}\right)e^{\mathrm{il}\phi_{\tilde{p}}}$ was the Fourier spectrum with a helical phase. The probability density of Volkov‐Bessel state was calculated as $\rho^{s,l}\;\left(x\right)=\psi_{p_{⊥},p_{z}}^{s,l†}\;\psi_{p_{⊥},p_{z}}^{s,l}$. The transverse current density was given by $j_{⊥}^{s,l}\;\left(x\right)=\psi_{p_{⊥},p_{z}}^{s,l†}\;\alpha_{⊥}\psi_{p_{⊥},p_{z}}^{s,l}$.

The photon QVS, $A_{k_{⊥},k_{z};\mu }^{\lambda ,j}\left(x\right)$, was constructed in a similar way using the plane‐wave state,^[^ ^16^, ^40^ ^]^ which was labeled by energy *ω*, momenta *k*_⊥_ and *k_z_*, helicity λ  =   ± 1, and TAM projection *j* along *z*‐axis.

### S‐Matrix of the Vortex NCS Process

According to the QED theory, the S‐matrix of the NV→V+V process is

(5) $$\begin{array}{c} \;S_{fi}^{V}=\;-ie\int d^{4}x{\bar{\psi }}_{p_{⊥},p_{z}}^{s,l}\left(x\right)\gamma^{\mu }A_{k_{⊥},k_{z};\mu }^{\lambda ,j*}\left(x\right)\Psi_{Q}^{\sigma }\left(x\right) \end{array}$$

where $\Psi_{Q}^{\sigma }\left(x\right)$ is the incoming non‐vortex electron state and is considered as a wave packet form of the Volkov state, $\Psi_{Q}^{\sigma }\;\left(x\right)=\frac{1}{{\left(2\pi \right)}^{3/2}}\;\int d^{3}q\rho \left(q_{⊥},q_{z}\right)\psi_{q}^{\sigma }\left(x\right)$, with the weighting function ρ(*q*_⊥_,*q_z_*) and central energy‐momentum *E_Q_* and *Q_z_*, $A_{k_{⊥},k_{z};\mu }^{\lambda ,j}\left(x\right)$ is the QVS of emitted γ‐photon, $\psi_{p_{⊥},p_{z}}^{s,l}\left(x\right)$ is the QVS of the scattered electron. Suppose the laser pulse carrying a helicity of λ_0_ =  ± 1 propagates along *z*‐axis, *a* ^μ^ (ζ) = *a*_0_ (0,*cos*ζ, λ_0_ *sin*ζ,0), and it has a square envelope ζ  = ω_0_ (*t* − *z*) ∈ [0,  2*m*π] with duration *m* = 10 periods. The S‐matrix can be approximately derived if ρ(*q*_⊥_,*q_z_*) was considered as a narrow Gaussian distribution,

(6) $$\begin{array}{c} \begin{array}{c} S_{fi}^{V}∼\int \frac{dq_{z}}{\left|q_{z}\right|}\delta \left(E_{p}+\omega -E_{q}-p_{z}-k_{z}+q_{z}\right)\left(e^{ig\left(q_{z}\right)}-1\right)\rho_{z}\left(q_{z}\right)\xi^{s†}\Xi_{k,p}^{j,l}\left(q_{z}\right)\xi^{\sigma } \end{array}\;\;\;\; \end{array}$$

The delta function enforces the conservation of energy and longitudinal momentum. The phase factor $g\left(q_{z}\right)=m\pi \left({\tilde{E}}_{p}+\omega -{\tilde{E}}_{q}+{\tilde{p}}_{z}+k_{z}-{\tilde{q}}_{z}\right)/\omega_{0}$, quantities with tildes were the electron quasi‐energy and quasi‐momentum in the external CP laser field, satisfying ${\tilde{E}}_{p}^{2}-p_{⊥}^{2}-\;{\tilde{p}}_{z}^{2}={\tilde{E}}_{Q}^{2}\;-\;{\tilde{Q}}_{z}^{2}=M^{2}\;\left(1+a_{0}^{2}\right)$. $\Xi_{k,p}^{j,l}\left(q_{z}\right)$ was a 2 × 2 matrix, its four elements were linked to the spin polarization of incoming and outgoing electrons.

To calculate the V→V+V process, the incoming electron state in Equation (5) was replaced with a wave packet of the Volkov‐Bessel state.

The narrow wave packet approximation in the longitudinal direction was used in the calculations, which means the longitudinal width of the electron wave packet was much smaller than its central value *Q_z_*.

The detailed derivation of the S‐matrix is shown in the Supporting Information.

### Vortex and Non‐Vortex Bases

The AMs of the scattered electron and γ‐photon were entangled and governed by the TAM conservation.^[^ ^41^, ^42^ ^]^ Once the AM of one particle were measured, the one of the other was also known. The QVS was the eigenstate of TAM operator, so it was more straightforward to study the AM features under the vortex basis. Using Equation (4) the S‐matrix, Equation (5), can be represented by the coherent superposition of the non‐vortex S‐matrix with helical phases of the final particles,

(7) $$\begin{array}{c} \;S_{fi}^{V}=\frac{i^{-l-j}}{2\pi }\;\int d\phi_{p}d\phi_{k}e^{-il\phi_{p}-ij\phi_{k}}S_{fi}^{NV}\left(\sigma ,Q\to s,p;\lambda ,k\right) \end{array}$$

Similarly, the non‐vortex S‐matrix can also be represented in the vortex basis,

(8) $$\begin{array}{c} \;S_{fi}^{NV}=\frac{1}{2\pi }\;\sum_{l,j}i^{-l-j}e^{il\phi_{p}+ij\phi_{k}}S_{fi}^{V}\left(\sigma ,Q\to s,l,p_{⊥},p_{z};\lambda ,j,k_{⊥},k_{z}\right) \end{array}$$

Theoretically, it was possible to attain OAM information when describing the final particles with the non‐vortex states. This requires the coherent superposition of the non‐vortex scattering matrix with helical phases according to Equation (7), which actually equates to a transformation from the non‐vortex basis to the vortex basis.

### Parameter *κ* versus the QED Parameter ***χ***_0_

The parameter *κ* can be correlated with the Lorentz‐invariant quantum parameter χ_0_ = (*E*_0_/*E_cr_*)(*k*_0_ · *Q*)/(ω_0_ *M*)  as: $\chi_{0}\approx \;\left(a_{0}^{3}/N\right)\kappa /\left(1-\kappa \right)=\left(a_{0}^{3}/N\right)\;\left(l_{c}/j_{c}\right)$. At the turning point *κ* = 0.5, it got *l_c_* = *j_c_*, and the quantum parameter $\chi_{0}\approx a_{0}^{3}/N$. When the laser amplitude *a*_0_ = 1, the number of absorbed laser photons is *N* = 25 and the quantum parameter $\chi_{0}∼0.04$ at the turning point so that the quantum effects were negligible. As the laser amplitude increases to 10 and 100, *N* at the turning point increases to 1700 and 1.65 × 10^5^, respectively, leading to larger values of χ_0_ (0.59 and 6, respectively) and more significant quantum effects.

## Conflict of Interest

The authors declare no conflict of interest.

## Supporting information

Supporting Information

## Data Availability

All data needed to evaluate the conclusions of the paper are present in the paper.
