# Supplementary material for: Generation of Quantum Vortex Electrons with Intense Laser Pulses
Source: Adv Sci (Weinh). 2024 Sep 3;11(41):2404564. doi: 10.1002/advs.202404564 (PMC11538682; doi:10.1002/advs.202404564)
Supplement: Supplementary file 1 — Supporting Information [file ADVS-11-2404564-s001.pdf]

ADVANCED SCIENCE
Open Access

Supporting Information

for *Adv. Sci.*, DOI 10.1002/advs.202404564

Generation of Quantum Vortex Electrons with Intense Laser Pulses

Zhigang Bu, Liangliang Ji, Xuesong Geng, Shiyu Liu, Shaohu Lei, Baifei Shen, Ruxin Li and Zhizhan Xu*

Supporting Information

Generation of Quantum Vortex Electrons with Intense Laser Pulses

Zhigang Bu, Liangliang Ji, Xuesong Geng, Shiyu Liu, Shaohu Lei, Baifei Shen, Ruxin Li, and Zhizhan Xu*

I. Mean Value of Electron AM Projection and Evolution of Volkov-Bessel State

The Volkov-Bessel state of electron in a laser field is constructed by coherently superposing the Volkov states, $\psi^s_{\tilde{p}}(x)$, over their azimuthal angles, $\psi^{s,l}_{p_\perp,p_z}(x)=\int d\tilde{p}_z d\phi_{\tilde{p}} d\tilde{p}_\perp \tilde{p}_\perp f^l_{p_\perp,p_z}(\tilde{\boldsymbol{p}})\psi^s_{\tilde{\boldsymbol{p}}}(x)$ with the Fourier spectrum $f^l_{p_\perp,p_z}(\tilde{\boldsymbol{p}})=\left(1/\sqrt{2\pi}i^l\tilde{p}_\perp\right)\delta(\tilde{p}_\perp-p_\perp)\delta(\tilde{p}_z-p_z)e^{il\phi_{\tilde{p}}}$. This state is not an eigenstate of any angular momentum projection. Here we derive the mean values of electron OAM and SAM projections onto the z-axis in a circularly polarized (CP) laser field. Using the Volkov-Bessel state, $\psi^{s,l}_{p_\perp,p_z}$, the electron OAM projection is calculated by

$$\langle L_z\rangle=\frac{\left\langle\psi^{s,l}_{p_\perp,p_z}\right|\left(-i\partial_\theta\right)\left|\psi^{s,l}_{p_\perp,p_z}\right\rangle}{\left\langle\psi^{s,l}_{p_\perp,p_z}\middle|\psi^{s,l}_{p_\perp,p_z}\right\rangle}=l+\Delta l\,, \tag{S1}$$

$$\Delta l=\frac{p_\perp^2}{2|\boldsymbol{p}|^2}\left(1-\frac{M}{E_p}\right)\left[\bar{s}_z+\frac{a_0^2M^2\omega_0\left(E_p+M\right)}{\left(k_0\cdot p\right)^2}\left(\lambda_0+\frac{\bar{s}_z\omega_0}{2\left(E_p+M\right)}\right)\right]\left(1+\frac{a_0^2M^2\omega_0}{2E_p\left(k_0\cdot p\right)}\right)^{-1}. \tag{S2}$$

Here $\bar{s}_z=\xi^{s\dagger}\sigma_z\xi^s/\xi^{s\dagger}\xi^s$ is the mean values of electron SAM under the free spinor ξ^s. The electron SAM projection is given by

$$\langle S_z\rangle=\frac{\left\langle\psi^{s,l}_{p_\perp,p_z}\right|\left(\Sigma_z/2\right)\left|\psi^{s,l}_{p_\perp,p_z}\right\rangle}{\left\langle\psi^{s,l}_{p_\perp,p_z}\middle|\psi^{s,l}_{p_\perp,p_z}\right\rangle}=\frac{\bar{s}_z}{2}+\Delta s\,, \tag{S3}$$

$$\Delta s=-\frac{\bar{s}_z}{2}\left[\frac{p_\perp^2}{|\boldsymbol{p}|^2}\left(1-\frac{M}{E_p}\right)\left(1-\frac{a_0^2M^2\omega_0^2}{2\left(k_0\cdot p\right)^2}\right)+\frac{a_0^2M^2\omega_0}{E_p\left(k_0\cdot p\right)}\right]\left(1+\frac{a_0^2M^2\omega_0}{2E_p\left(k_0\cdot p\right)}\right)^{-1}. \tag{S4}$$

The mean values of electron TAM projection is obtained from Equations (S1) and (S3),

$$\langle J_z\rangle=\langle L_z+S_z\rangle=l+\frac{\bar{s}_z}{2}+\Delta j\,, \tag{S5}$$

$$\Delta j = -\frac{a_0^2 M^2 \omega_0}{2E_p (k\cdot p)}\left[\bar{s}_z - \frac{p_\perp^2}{(k_0\cdot p)}\left(\lambda_0 + \frac{\bar{s}_z \omega_0 (E_p - M)}{|\boldsymbol{p}|^2}\right)\right]\left(1+\frac{a_0^2 M^2 \omega_0}{2E_p (k_0\cdot p)}\right)^{-1} \quad \text{(S6)}$$

When the laser field vanishes, $a_0 \to 0$, we get $\Delta l \to (\bar{s}_z p_\perp^2/2\boldsymbol{p}^2)(1 - M/E_p)$, $\Delta s \to -(\bar{s}_z p_\perp^2/2\boldsymbol{p}^2)(1 - M/E_p)$, and $\Delta j \to 0$, which is in agreement with the quantum vortex state (QVS) of free electron.

The probability density of Volkov-Bessel state is

$$\begin{aligned}
\rho^{s,l}(x) &= \left(\psi^{s,l}_{p_\perp,p_z}\right)^\dagger \psi^{s,l}_{p_\perp,p_z} \\
&\sim \frac{E_p - M}{2E_p|\boldsymbol{p}|^2}\left\{\left[\left((E_p+M)^2 + p_z^2\right) + \frac{a_0^2 M^2 \omega_0^2 (E_p + M - p_z)^2}{2(k_0\cdot p)^2}\right]|\mathcal{I}_l|^2\right. \\
&\quad \left. - \frac{\lambda_0 a_0 M \omega_0 p_\perp (E_p+M)}{(k_0\cdot p)}\left(\mathcal{I}_l \mathcal{I}^*_{l+s} + \mathcal{I}_l^* \mathcal{I}_{l+s}\right) + p_\perp^2\left(1+\frac{a_0^2 M^2 \omega_0^2}{2(k_0\cdot p)^2}\right)|\mathcal{I}_{l+s}|^2\right\},
\end{aligned} \quad \text{(S7)}$$

$$\mathcal{I}_\eta = \sum_n i^n \lambda_0^n e^{in(\theta - \lambda_0 \zeta)} J_n(p_\perp r) J_{\eta+n}\left(\frac{a_0 M p_\perp}{(k_0\cdot p)}\right). \quad \text{(S8)}$$

When electron runs out of the laser field, its wave function simplifies to the free Bessel state,

$$\begin{aligned}
\psi^{s,l}_{p_\perp,p_z}(a_0 = 0) &= \frac{e^{ip_z z - iE_p t}}{(2\pi)|\boldsymbol{p}|}\sqrt{\frac{E_p - M}{2E_p}}\left[\begin{pmatrix}(E_p+M)\xi^s \\ p_z\sigma_z\xi^s\end{pmatrix} J_l(p_\perp r)e^{il\theta} + ip_\perp\begin{pmatrix}0 \\ \sigma_\perp \xi^s\end{pmatrix}\right], \\
\sigma_\perp &= \begin{pmatrix} 0 & -J_{l-1}(p_\perp r)e^{i(l-1)\theta} \\ J_{l+1}(p_\perp r)e^{i(l+1)\theta} & 0\end{pmatrix}.
\end{aligned} \quad \text{(S9)}$$

Its probability density simplifies to the form,

$$\rho^{s,l}(a_0 \to 0) \sim \left[\left(1+\frac{M}{E_p}\right) + \left(1-\frac{M}{E_p}\right)\frac{p_z^2}{|\boldsymbol{p}|^2}\right] J_l^2(p_\perp r) + \frac{p_\perp^2}{|\boldsymbol{p}|^2}\left(1-\frac{M}{E_p}\right) J_{l+s}^2(p_\perp r). \quad \text{(S10)}$$

The transverse current density of electron Volkov-Bessel state is

$$\begin{aligned}
\boldsymbol{j}_\perp^{s,l}(x) &= \left(\psi^{s,l}_{p_\perp,p_z}\right)^\dagger \boldsymbol{\alpha}_\perp \psi^{s,l}_{p_\perp,p_z} \\
&\sim -\frac{E_p - M}{E_p|\boldsymbol{p}|^2}\left\{\lambda_0 p_\perp (E_p+M)\left[\mathrm{Re}\left(\mathcal{I}_l\mathcal{I}^*_{l+s}e^{i\lambda_0 s\zeta}\right)\boldsymbol{e}_x + s\,\mathrm{Im}\left(\mathcal{I}_l\mathcal{I}^*_{l+s}e^{i\lambda_0 s\zeta}\right)\boldsymbol{e}_y\right]\right. \\
&\quad \left. - \frac{a_0 M \omega_0}{2(k_0\cdot p)}\left[(E_p + M - p_z)^2|\mathcal{I}_l|^2 + p_\perp^2|\mathcal{I}_{l+s}|^2\right]\left(\cos\zeta\,\boldsymbol{e}_x + \lambda_0 \sin\zeta\,\boldsymbol{e}_y\right)\right\},
\end{aligned} \quad \text{(S11)}$$

The first term is the intrinsic current vector of Volkov-Bessel state, describing the vortex structure of an electron. The second term is the extrinsic current vector driven by the laser field. In the limit of $a_0 \to 0$, the extrinsic current vanishes, while intrinsic current simplifies to

$$\boldsymbol{j}_\perp^{s,l}(a_0 \to 0) \sim -\frac{p_\perp}{E_p} J_l(p_\perp r) J_{l+s}(p_\perp r)\left(\sin\theta\,\boldsymbol{e}_x - \cos\theta\,\boldsymbol{e}_y\right). \quad \text{(S12)}$$

The above discussion indicates that the free electron QVS is stable, its density probability and current vector do not evolve with time in free space. The non-vortex electron state in laser field is the Volkov state. When propagating into a free space, it simplifies to a plane-wave state, and does not evolve either.

II. NV→V+V scattering

Here we obtain the S-matrix and scattered rate of the nonlinear Compton scattering (NCS) from a non-vortex high-energy electron into a QVS electron and a γ-photon in a CP laser field. We use a wave packet to describe the incoming electron, its weighting function is defined as Gaussian distribution in transverse and longitudinal directions: $\rho(q_\perp, q_z) = \rho_\perp(q_\perp)\rho_z(q_z) = N_{\tau_\perp,\tau_z} exp[-4q_\perp^2/\tau_\perp^2 - 4(q_z - Q_z)^2/\tau_z^2]$, with the normalized factor $N_{\tau_\perp,\tau_z}$. The S-matrix takes the form, $S_{fi}^V = -ie\int d^4x\bar{\psi}_{p_\perp,p_z}^{s,l}(x)\gamma^\mu A_{k_\perp,k_z;\mu}^{\lambda,j*}(x)\Psi_Q^\sigma(x)$. We consider the laser pulse has a square envelope for convenience, under the narrow-wave-packet approximation in longitudinal direction, the S-matrix can be simplified into,

$$\begin{aligned} S_{fi}^V \approx & \frac{i^{l-j}e(-\lambda_0)^{j+l} N_{\tau_\perp,\tau_z}\tau_\perp^2}{16(2\pi)^2|\boldsymbol{p}|}\sqrt{\frac{(E_Q-M)(E_p-M)}{E_Q E_p\omega}}\exp\left[-\frac{a_0^2M^2\tau_\perp^2}{16(k_0\cdot Q)^2}\right] \\ & \times\int\frac{dq_z}{|q_z|}\exp\left[-\frac{4(q_z-Q_z)^2}{\tau_z^2}\right]\left[\exp\left(\frac{im\pi}{\omega_0}\left(\tilde{E}_p+\omega-\tilde{E}_q+\tilde{p}_z+k_z-\tilde{q}_z\right)\right)-1\right] \\ & \times\delta\left(E_p+\omega-E_q-p_z-k_z+q_z\right)\xi^{s\dagger}\Xi_{k,p}^{j,l}(q_z)\xi^\sigma. \end{aligned} \tag{S13}$$

The four matrix elements of $\Xi_{k,p}^{j,l}$ is given by,

$$\begin{aligned} \upsilon_{11} \approx & \frac{1}{W_0}\sum_n\left\{\left[-\frac{1}{\left(E_p+\omega-E_q+p_z+k_z-q_z\right)}\left(\frac{a_0^2M^2\omega_0}{(k_0\cdot p)}-\frac{a_0^2M^2\omega_0}{(k_0\cdot Q)}-2(j+l)\lambda_0\omega_0\right)\right.\right. \\ & \times\left[\frac{\lambda k_\perp}{\omega}\left(q_z\left(E_p+M\right)+p_z\left(E_q+M\right)\right)R_{j+n,p_\perp}^{j,k_\perp}+p_\perp\left(E_q+M\right)\left(1+\frac{\lambda k_z}{\omega}\right)R_{j+n-1,p_\perp}^{j-1,k_\perp}\right] \\ & \left.+\frac{\lambda a_0^2M^2\omega_0^2k_\perp}{2\omega(k_0\cdot Q)(k_0\cdot p)}\left(E_q+M-q_z\right)\left(E_p+M-p_z\right)R_{j+n,p_\perp}^{j,k_\perp}\right]J_{j+l+n}\left(\frac{a_0Mp_\perp}{(k_0\cdot p)}\right) \\ & +\frac{\lambda_0a_0M\omega_0}{2}\left[\frac{\left(E_q+M-q_z\right)\left(E_p+M-p_z\right)}{(k_0\cdot Q)}\left(1-\frac{\lambda k_z}{\omega}\right)R_{j+n+1,p_\perp}^{j+1,k_\perp}\right. \\ & \left.-\frac{\lambda k_\perp p_\perp}{\omega}\left(\frac{\left(E_q+M-q_z\right)}{(k_0\cdot Q)}+\frac{\left(E_q+M+q_z\right)}{(k_0\cdot p)}\right)R_{j+n,p_\perp}^{j,k_\perp}\right]J_{j+l+n+1}\left(\frac{a_0Mp_\perp}{(k_0\cdot p)}\right) \\ & \left.-\frac{\lambda_0a_0M\omega_0}{2(k_0\cdot p)}\left(1+\frac{\lambda k_z}{\omega}\right)\left(E_q+M-q_z\right)\left(E_p+M-p_z\right)R_{j+n-1,p_\perp}^{j-1,k_\perp}J_{j+l+n-1}\left(\frac{a_0Mp_\perp}{(k_0\cdot p)}\right)\right\}, \end{aligned} \tag{S14a}$$

$$
\begin{aligned}
\upsilon_{12} \approx \frac{1}{W_1}\sum_n \Bigg\{ \Bigg[& \frac{\lambda_0}{\left(E_p+\omega-E_q+p_z+k_z-q_z\right)} \left(\frac{a_0^2 M^2 \omega_0}{(k_0\cdot p)} - \frac{a_0^2 M^2\omega_0}{(k_0\cdot Q)} - 2\left(j+l+1\right)\lambda_0\omega_0 \right) \\
& \times \left[\left(q_z\left(E_p+M\right) - p_z\left(E_q+M\right)\right)\left(1-\frac{\lambda k_z}{\omega}\right) R_{j+n+1,p_\perp}^{j+1,k_\perp} - \frac{\lambda k_\perp p_\perp}{\omega}\left(E_q+M\right) R_{j+n,p_\perp}^{j,k_\perp} \right] \\
& - \frac{\lambda_0 \lambda a_0^2 M^2 \omega_0^2 k_\perp p_\perp}{2\omega\left(k_0\cdot Q\right)\left(k_0\cdot p\right)} \left(E_q+M-q_z\right) R_{j+n,p_\perp}^{j,k_\perp} \Bigg] J_{j+l+n+1}\left(\frac{a_0 M p_\perp}{(k_0\cdot p)}\right) \\
& + \frac{a_0 M\omega_0}{2}\left[\frac{\lambda k_\perp}{\omega}\left(\frac{\left(E_q+M-q_z\right)\left(E_p+M+p_z\right)}{(k_0\cdot Q)} - \frac{\left(E_q+M+q_z\right)\left(E_p+M-p_z\right)}{(k_0\cdot p)} \right) \right. \\
& \left. \times R_{j+n,p_\perp}^{j,k_\perp} + \frac{p_\perp}{(k_0\cdot Q)}\left(1+\frac{\lambda k_z}{\omega}\right)\left(E_q+M-q_z\right) R_{j+n-1,p_\perp}^{j-1,k_\perp} \right] J_{j+l+n}\left(\frac{a_0 M p_\perp}{(k_0\cdot p)}\right) \\
& - \frac{a_0 M \omega_0 p_\perp}{2\left(k_0\cdot p\right)}\left(1-\frac{\lambda k_z}{\omega}\right)\left(E_q+M-q_z\right) R_{j+n+1,p_\perp}^{j+1,k_\perp} J_{j+l+n+2}\left(\frac{a_0 M p_\perp}{(k_0\cdot p)}\right) \Bigg\},
\end{aligned}
\tag{S14b}
$$

$$
\begin{aligned}
\upsilon_{21} \approx \frac{1}{W_{-1}}\sum_n \Bigg\{ \Bigg[& \frac{\lambda_0}{\left(E_p+\omega-E_q+p_z+k_z-q_z\right)} \left(\frac{a_0^2 M^2 \omega_0}{(k_0\cdot p)} - \frac{a_0^2 M^2\omega_0}{(k_0\cdot Q)} - 2\left(j+l-1\right)\lambda_0\omega_0 \right) \\
& \times \left[\left(q_z\left(E_p+M\right) - p_z\left(E_q+M\right)\right)\left(1+\frac{\lambda k_z}{\omega}\right) R_{j+n-1,p_\perp}^{j-1,k_\perp} + \frac{\lambda k_\perp p_\perp}{\omega}\left(E_q+M\right) R_{j+n,p_\perp}^{j,k_\perp} \right] \\
& + \frac{\lambda_0 \lambda a_0^2 M^2 \omega_0^2 k_\perp p_\perp}{2\omega\left(k_0\cdot Q\right)\left(k_0\cdot p\right)} \left(E_q+M-q_z\right) R_{j+n,p_\perp}^{j,k_\perp} \Bigg] J_{j+l+n-1}\left(\frac{a_0 M p_\perp}{(k_0\cdot p)}\right) \\
& + \frac{a_0 M\omega_0}{2}\left[\frac{\lambda k_\perp}{\omega}\left(\frac{\left(E_q+M+q_z\right)\left(E_p+M-p_z\right)}{(k_0\cdot p)} - \frac{\left(E_q+M-q_z\right)\left(E_p+M+p_z\right)}{(k_0\cdot Q)} \right) \right. \\
& \left. \times R_{j+n,p_\perp}^{j,k_\perp} + \frac{p_\perp}{(k_0\cdot Q)}\left(1-\frac{\lambda k_z}{\omega}\right)\left(E_q+M-q_z\right) R_{j+n+1,p_\perp}^{j+1,k_\perp} \right] J_{j+l+n}\left(\frac{a_0 M p_\perp}{(k_0\cdot p)}\right) \\
& - \frac{a_0 M \omega_0 p_\perp}{2\left(k_0\cdot p\right)}\left(1+\frac{\lambda k_z}{\omega}\right)\left(E_q+M-q_z\right) R_{j+n-1,p_\perp}^{j-1,k_\perp} J_{j+l+n-2}\left(\frac{a_0 M p_\perp}{(k_0\cdot p)}\right) \Bigg\},
\end{aligned}
\tag{S14c}
$$

$$\upsilon_{22} \approx \frac{1}{W_0}\sum_n \Bigg\{ \Bigg[\frac{1}{\left(E_p+\omega-E_q+p_z+k_z-q_z\right)} \left(\frac{a_0^2 M^2 \omega_0}{(k_0 \cdot p)} - \frac{a_0^2 M^2 \omega_0}{(k_0 \cdot Q)} - 2(j+l)\lambda_0\omega_0 \right)$$
$$\times \left[p_\perp \left(E_q+M\right)\left(1-\frac{\lambda k_z}{\omega}\right) R^{j+1,k_\perp}_{j+n+1,p_\perp} - \frac{\lambda k_\perp}{\omega}\left(q_z\left(E_p+M\right)+p_z\left(E_q+M\right)\right) R^{j,k_\perp}_{j+n,p_\perp} \right]$$
$$+\frac{\lambda a_0^2 M^2 \omega_0^2 k_\perp}{2\omega (k_0\cdot Q)(k_0\cdot p)}\left(E_q+M-q_z\right)\left(E_p+M-p_z\right) R^{j,k_\perp}_{j+n,p_\perp} \Bigg] J_{j+l+n}\left(\frac{a_0 M p_\perp}{(k_0\cdot p)}\right)$$
$$-\frac{\lambda_0 a_0 M \omega_0}{2}\Bigg[\frac{\left(E_q+M-q_z\right)\left(E_p+M-p_z\right)}{(k_0\cdot Q)}\left(1+\frac{\lambda k_z}{\omega}\right) R^{j-1,k_\perp}_{j+n-1,p_\perp} \tag{S14d}$$
$$+\frac{\lambda k_\perp p_\perp}{\omega}\left(\frac{\left(E_q+M-q_z\right)}{(k_0\cdot q)} + \frac{\left(E_q+M+q_z\right)}{(k_0\cdot p)} \right) R^{j,k_\perp}_{j+n,p_\perp} \Bigg] J_{j+l+n-1}\left(\frac{a_0 M p_\perp}{(k_0\cdot p)}\right)$$
$$+\frac{\lambda_0 a_0 M \omega_0}{2(k_0\cdot p)}\left(1-\frac{\lambda k_z}{\omega}\right)\left(E_q+M-q_z\right)\left(E_p+M-p_z\right) R^{j+1,k_\perp}_{j+n+1,p_\perp} J_{j+l+n+1}\left(\frac{a_0 M p_\perp}{(k_0\cdot p)}\right)\Bigg\}.$$

Here $J_s(x)$ is the Bessel function of the first kind, $W_\Delta = \tilde{E}_p + \omega - \tilde{E}_q + \tilde{p}_z + k_z - \tilde{q}_z - 2(j+l+\Delta)\lambda_0\omega_0$, function $R^{j+\Delta}_{j+n+\Delta}$ takes the form,

$$R^{j+\Delta}_{j+n+\Delta}\left(k_\perp, p_\perp\right) = \frac{16}{\tau_\perp^2}\int_0^\infty dx x \exp\left(-x^2\right) J_{j+\Delta}\left(\frac{4k_\perp}{\tau_\perp}x\right) J_{j+n+\Delta}\left(\frac{4p_\perp}{\tau_\perp}x\right) I_{|n|}\left(\alpha x\right), \tag{S15}$$

with $\Delta = 0, \pm 1$, $x = \tau_\perp r/4$, $\alpha = a_0 M \tau_\perp / 2(k_0 \cdot Q)$, and the modified Bessel function $I_s(x)$.

Using the S-matrix, Equation (S13), one obtains the AM-dependent differential scattering rate, $dP = \left|S^V_{fi}\right|^2 p_\perp dp_\perp dp_z k_\perp dk_\perp dk_z$. Electron polarization properties are implied in the matrix elements of $\Xi^{j,l}_{k,p}$ and determined by the basis spinors of incoming and scattered electrons, ξ^σ and ξ^s. Assuming that the incoming electron are polarized in +z direction in the free frame. If ξ^σ is the eigen-mode of spin z-projection with the eigen-value +1: $\sigma_z \xi^\sigma = \xi^\sigma$, the polarized scattering rate can be derived by choosing the polarization state of scattered electron ξ^s. If the scattered electron carries the same spin polarization as the incoming electron, $\sigma_z \xi^s = \xi^s$, the scattered rate is determined by the trace of $\mathrm{Tr}\left[\xi^\sigma \xi^{\sigma\dagger} \Xi^\dagger \xi^s \xi^{s\dagger} \Xi\right] = \left|\upsilon_{11}\right|^2$. The spin-flip scattered electron leads to the trace of $\mathrm{Tr}\left[\xi^\sigma \xi^{\sigma\dagger} \Xi^\dagger \xi^s \xi^{s\dagger} \Xi\right] = \left|\upsilon_{21}\right|^2$. For incoming electron polarized in –z direction, $\left|\upsilon_{12}\right|^2$ and $\left|\upsilon_{22}\right|^2$ formulate the rates of spin-flip and non-flip scattered electrons. That means the four matrix elements in $\Xi^{j,l}_{k,p}$ determine the scattering rates of electrons in different spin-channels.

III. V→V+V scattering

To describe the scattering of a QVS electron with SAM and OAM numbers $\tilde{s}$ and $\tilde{l}$, the S-matrix can be derived by replacing the Volkov state with the Volkov-Bessel state for the incoming electron state. We also consider the incoming electron takes wave packet form with weighting function $\rho(q_\perp, q_z) = N_{Q,\tau_\perp,\tau_z} \exp\left[-4(q_\perp - Q_\perp)^2/\tau_\perp^2 - 4(q_z - Q_z)^2/\tau_z^2\right]$. Under the narrow-wave-packet approximation the S-matrix is given by

$$S_{fi} \approx \frac{i^{l-\tilde{l}-j} e(-\lambda_0)^{j+l+\tilde{l}} N_{Q,\tau_\perp,\tau_z}}{2(2\pi)^3 |\boldsymbol{p}|} \sqrt{\frac{(E_Q - M)(E_p - M)}{E_Q E_p \omega}} \int \frac{dq_z}{|\boldsymbol{q}|} \exp\left[-\frac{4(q_z - Q_z)^2}{\tau_z^2}\right] \xi^{s\dagger} \Xi'^{j,l}_{k,p}(q_z) \xi^{\sigma}$$
$$\times \left[\exp\left(\frac{im\pi}{\omega_0}\left(\tilde{E}_p + \omega - \tilde{E}_q + \tilde{p}_z + k_z - \tilde{q}_z\right)\right) - 1\right] \delta\left(E_p + \omega - E_q - p_z - k_z + q_z\right)$$

(S16)

with the four matrix elements of $\Xi'^{j,l}_{k,p}$,

$$\upsilon'_{11} \approx \frac{1}{W'_0} \sum_n \Bigg\{ \Bigg[\Bigg[-\frac{1}{(E_p + \omega - E_Q + p_z + k_z - Q_z)} \left(\frac{a_0^2 M^2 \omega_0}{(k_0 \cdot p)} - \frac{a_0^2 M^2 \omega_0}{(k_0 \cdot Q)} - 2(j + l - \tilde{l}) \lambda_0 \omega_0 \right)$$
$$\times \left[\frac{\varsigma_0^j \lambda k_\perp}{\omega} \left(p_z (E_Q + M) + Q_z (E_p + M) \right) + \varsigma_0^{j-1} p_\perp (E_Q + M) \left(1 + \frac{\lambda k_z}{\omega} \right) \right]$$
$$+ \frac{\varsigma_0^j \lambda a_0^2 M^2 \omega_0^2 k_\perp}{2\omega (k_0 \cdot Q)(k_0 \cdot p)} (E_Q + M - Q_z)(E_p + M - p_z) \Bigg] J_{j+l+n}\left(\frac{a_0 M p_\perp}{(k_0 \cdot p)} \right)$$
$$+ \frac{\lambda_0 a_0 M \omega_0}{2} \Bigg[\frac{\varsigma_0^{j+1}}{(k_0 \cdot Q)} \left(1 - \frac{\lambda k_z}{\omega} \right) (E_Q + M - Q_z)(E_p + M - p_z)$$
$$- \frac{\varsigma_0^j \lambda k_\perp p_\perp}{\omega} \left(\frac{(E_Q + M - Q_z)}{(k_0 \cdot Q)} + \frac{(E_Q + M + Q_z)}{(k_0 \cdot p)} \right) \Bigg] J_{j+l+n+1}\left(\frac{a_0 M p_\perp}{(k_0 \cdot p)} \right)$$
$$- \frac{\varsigma_0^{j-1} \lambda_0 a_0 M \omega_0}{2(k_0 \cdot p)} \left(1 + \frac{\lambda k_z}{\omega} \right) (E_Q + M - Q_z)(E_p + M - p_z) J_{j+l+n-1}\left(\frac{a_0 M p_\perp}{(k_0 \cdot p)} \right) \Bigg]$$
$$- \Bigg[\frac{\lambda_0 a_0 M \omega_0}{2} \left[\frac{\varsigma_1^j \lambda k_\perp}{\omega} \left(\frac{(E_p + M - p_z)}{(k_0 \cdot p)} + \frac{(E_p + M + p_z)}{(k_0 \cdot Q)} \right) + \frac{\varsigma_1^{j-1} p_\perp}{(k_0 \cdot Q)} \left(1 + \frac{\lambda k_z}{\omega} \right) \right]$$
$$\times J_{j+l+n}\left(\frac{a_0 M p_\perp}{(k_0 \cdot p)} \right) - \Bigg[\frac{\varsigma_1^{j+1} (E_p + M)}{(E_p + \omega - E_Q + p_z + k_z - Q_z)} \left(1 - \frac{\lambda k_z}{\omega} \right)$$
$$\times \left(\frac{a_0^2 M^2 \omega_0}{(k_0 \cdot p)} - \frac{a_0^2 M^2 \omega_0}{(k_0 \cdot Q)} - 2(j + l - \tilde{l}) \lambda_0 \omega_0 \right) + \frac{\varsigma_1^j \lambda a_0^2 M^2 \omega_0^2 k_\perp p_\perp}{2\omega (k_0 \cdot Q)(k_0 \cdot p)} \Bigg] J_{j+l+n+1}\left(\frac{a_0 M p_\perp}{(k_0 \cdot p)} \right)$$
$$- \frac{\varsigma_1^{j+1} \lambda_0 a_0 M \omega_0 p_\perp}{2(k_0 \cdot p)} \left(1 - \frac{\lambda k_z}{\omega} \right) J_{j+l+n+2}\left(\frac{a_0 M p_\perp}{(k_0 \cdot p)} \right) \Bigg] \Bigg\},$$

(S17a)

$$
\begin{aligned}
\upsilon_{12}' \approx \frac{1}{W_1'}\sum_n \Bigg\{ \Bigg[\Bigg[& \frac{\lambda_0}{\left(E_p+\omega-E_Q+p_z+k_z-Q_z\right)} \left(\frac{a_0^2 M^2 \omega_0}{(k_0\cdot p)} - \frac{a_0^2 M^2 \omega_0}{(k_0\cdot Q)} - 2\left(j+l-\tilde{l}+1\right)\lambda_0\omega_0 \right) \\
& \times \left[\varsigma_0^{j+1}\left(1-\frac{\lambda k_z}{\omega}\right)\left(Q_z\left(E_p+M\right)-p_z\left(E_Q+M\right)\right) - \frac{\varsigma_0^{j}\lambda k_\perp p_\perp}{\omega}\left(E_Q+M\right)\right] \\
& - \frac{\varsigma_0^{j}\lambda_0\lambda a_0^2 M^2\omega_0^2 k_\perp p_\perp}{2\omega(k_0\cdot Q)(k_0\cdot p)}\left(E_Q+M-Q_z\right)\Bigg] J_{j+l+n+1}\left(\frac{a_0 M p_\perp}{(k_0\cdot p)}\right) \\
& + \frac{a_0 M\omega_0}{2}\Bigg[\frac{\varsigma_0^{j}\lambda k_\perp}{\omega}\left(\frac{\left(E_Q+M-Q_z\right)\left(E_p+M+p_z\right)}{(k_0\cdot Q)} - \frac{\left(E_Q+M+Q_z\right)\left(E_p+M-p_z\right)}{(k_0\cdot p)}\right) \\
& + \frac{\varsigma_0^{j-1}p_\perp}{(k_0\cdot Q)}\left(1+\frac{\lambda k_z}{\omega}\right)\left(E_Q+M-Q_z\right)\Bigg] J_{j+l+n}\left(\frac{a_0 M p_\perp}{(k_0\cdot p)}\right) \\
& - \frac{\varsigma_0^{j+1}a_0 M\omega_0 p_\perp}{2(k_0\cdot p)}\left(1-\frac{\lambda k_z}{\omega}\right)\left(E_Q+M-Q_z\right) J_{j+l+n+2}\left(\frac{a_0 M p_\perp}{(k_0\cdot p)}\right)\Bigg] \\
& - \Bigg[\frac{a_0 M\omega_0}{2}\left[\frac{\varsigma_{-1}^{j}\lambda k_\perp p_\perp}{\omega}\left(\frac{1}{(k_0\cdot Q)}-\frac{1}{(k_0\cdot p)}\right) - \frac{\varsigma_{-1}^{j+1}}{(k_0\cdot Q)}\left(1-\frac{\lambda k_z}{\omega}\right)\left(E_p+M-p_z\right)\right] \\
& \times J_{j+l+n+1}\left(\frac{a_0 M p_\perp}{(k_0\cdot p)}\right) - \frac{\varsigma_{-1}^{j}\lambda_0\lambda k_\perp}{\omega}\Bigg[\frac{\left(E_p+M\right)}{\left(E_p+\omega-E_Q+p_z+k_z-Q_z\right)} \\
& \times \left(\frac{a_0^2 M^2 \omega_0}{(k_0\cdot p)} - \frac{a_0^2 M^2 \omega_0}{(k_0\cdot Q)} - 2\left(j+l-\tilde{l}+1\right)\lambda_0\omega_0 \right) + \frac{a_0^2 M^2\omega_0^2\left(E_p+M-p_z\right)}{2(k_0\cdot Q)(k_0\cdot p)}\Bigg] \\
& \times J_{j+l+n}\left(\frac{a_0 M p_\perp}{(k_0\cdot p)}\right) + \frac{\varsigma_{-1}^{j-1}a_0 M\omega_0}{2(k_0\cdot p)}\left(1+\frac{\lambda k_z}{\omega}\right)\left(E_p+M-p_z\right) J_{j+l+n-1}\left(\frac{a_0 M p_\perp}{(k_0\cdot p)}\right)\Bigg]\Bigg\},
\end{aligned}
\tag{S17b}
$$

$$
\begin{aligned}
\upsilon'_{21} \approx \frac{1}{W'_{-1}}\sum_{n}\Bigg\{\Bigg[\Bigg[&\frac{\lambda_0}{\left(E_p+\omega-E_Q+p_z+k_z-Q_z\right)}\left(\frac{a_0^2M^2\omega_0}{(k_0\cdot p)}-\frac{a_0^2M^2\omega_0}{(k_0\cdot Q)}-2\left(j+l-\tilde{l}-1\right)\lambda_0\omega_0\right)\\
&\times\left[\varsigma_0^{j-1}\left(1+\frac{\lambda k_z}{\omega}\right)\left(Q_z\left(E_p+M\right)-p_z\left(E_Q+M\right)\right)+\frac{\varsigma_0^{j}\lambda k_\perp p_\perp}{\omega}\left(E_Q+M\right)\right]\\
&+\frac{\varsigma_0^{j}\lambda_0\lambda a_0^2M^2\omega_0^2k_\perp p_\perp}{2\omega(k_0\cdot Q)(k_0\cdot p)}\left(E_Q+M-Q_z\right)\Bigg]J_{j+l+n-1}\left(\frac{a_0Mp_\perp}{(k_0\cdot p)}\right)\\
&+\frac{a_0M\omega_0}{2}\left[\frac{\varsigma_0^{j}\lambda k_\perp}{\omega}\left(\frac{\left(E_Q+M+Q_z\right)\left(E_p+M-p_z\right)}{(k_0\cdot p)}-\frac{\left(E_Q+M-Q_z\right)\left(E_p+M+p_z\right)}{(k_0\cdot Q)}\right)\right.\\
&\left.+\frac{\varsigma_0^{j+1}p_\perp}{(k_0\cdot Q)}\left(1-\frac{\lambda k_z}{\omega}\right)\left(E_Q+M-Q_z\right)\right]J_{j+l+n}\left(\frac{a_0Mp_\perp}{(k_0\cdot p)}\right)\\
&-\frac{\varsigma_0^{j-1}a_0M\omega_0p_\perp}{2(k_0\cdot p)}\left(1+\frac{\lambda k_z}{\omega}\right)\left(E_Q+M-Q_z\right)J_{j+l+n-2}\left(\frac{a_0Mp_\perp}{(k_0\cdot p)}\right)\Bigg]\\
&-\Bigg[\frac{a_0M\omega_0}{2}\left[\frac{\varsigma_1^{j}\lambda k_\perp p_\perp}{\omega}\left(\frac{1}{(k_0\cdot p)}-\frac{1}{(k_0\cdot Q)}\right)-\frac{\varsigma_1^{j-1}}{(k_0\cdot Q)}\left(1+\frac{\lambda k_z}{\omega}\right)\left(E_p+M-p_z\right)\right]\\
&\times J_{j+l+n-1}\left(\frac{a_0Mp_\perp}{(k_0\cdot p)}\right)+\frac{\varsigma_1^{j}\lambda_0\lambda k_\perp}{\omega}\left[\frac{\left(E_p+M\right)}{\left(E_p+\omega-E_Q+p_z+k_z-Q_z\right)}\right.\\
&\left.\times\left(\frac{a_0^2M^2\omega_0}{(k_0\cdot p)}-\frac{a_0^2M^2\omega_0}{(k_0\cdot Q)}-2\left(j+l-\tilde{l}-1\right)\lambda_0\omega_0\right)+\frac{a_0^2M^2\omega_0^2\left(E_p+M-p_z\right)}{2(k_0\cdot Q)(k_0\cdot p)}\right]\\
&\times J_{j+l+n}\left(\frac{a_0Mp_\perp}{(k_0\cdot p)}\right)+\frac{\varsigma_1^{j+1}a_0M\omega_0}{2(k_0\cdot p)}\left(1-\frac{\lambda k_z}{\omega}\right)\left(E_p+M-p_z\right)J_{j+l+n+1}\left(\frac{a_0Mp_\perp}{(k_0\cdot p)}\right)\Bigg]\Bigg\},
\end{aligned}
$$

(S17c)

$$
\begin{aligned}
\upsilon'_{22} \approx \frac{1}{W'_0}\sum_n \Bigg\{ \Bigg[\Bigg[& -\frac{1}{\left(E_p+\omega-E_Q+p_z+k_z-Q_z\right)}\left(\frac{a_0^2M^2\omega_0}{(k_0\cdot p)}-\frac{a_0^2M^2\omega_0}{(k_0\cdot Q)}-2\left(j+l-\tilde{l}\right)\lambda_0\omega_0\right) \\
& \times\left[\frac{\varsigma_0^j\lambda k_\perp}{\omega}\left(Q_z\left(E_p+M\right)+p_z\left(E_Q+M\right)\right)-\varsigma_0^{j+1}p_\perp\left(E_Q+M\right)\left(1-\frac{\lambda k_z}{\omega}\right)\right] \\
& +\frac{\varsigma_0^j\lambda a_0^2M^2\omega_0^2k_\perp}{2\omega(k_0\cdot Q)(k_0\cdot p)}\left(E_Q+M-Q_z\right)\left(E_p+M-p_z\right)\Bigg]J_{j+l+n}\left(\frac{a_0Mp_\perp}{(k_0\cdot p)}\right) \\
& -\frac{\lambda_0a_0M\omega_0}{2}\Bigg[\frac{\varsigma_0^{j-1}}{(k_0\cdot Q)}\left(1+\frac{\lambda k_z}{\omega}\right)\left(E_Q+M-Q_z\right)\left(E_p+M-p_z\right) \\
& +\frac{\varsigma_0^j\lambda k_\perp p_\perp}{\omega}\left(\frac{\left(E_Q+M-Q_z\right)}{(k_0\cdot Q)}+\frac{\left(E_Q+M+Q_z\right)}{(k_0\cdot p)}\right)\Bigg]J_{j+l+n-1}\left(\frac{a_0Mp_\perp}{(k_0\cdot p)}\right) \\
& +\frac{\varsigma_0^{j+1}\lambda_0a_0M\omega_0}{2(k_0\cdot p)}\left(1-\frac{\lambda k_z}{\omega}\right)\left(E_Q+M-Q_z\right)\left(E_p+M-p_z\right)J_{j+l+n+1}\left(\frac{a_0Mp_\perp}{(k_0\cdot p)}\right)\Bigg] \\
& -\Bigg[\frac{\lambda_0a_0M\omega_0}{2}\left[\frac{\varsigma_{-1}^j\lambda k_\perp}{\omega}\left(\frac{\left(E_p+M+p_z\right)}{(k_0\cdot Q)}+\frac{\left(E_p+M-p_z\right)}{(k_0\cdot p)}\right)-\frac{\varsigma_{-1}^{j+1}p_\perp}{(k_0\cdot Q)}\left(1-\frac{\lambda k_z}{\omega}\right)\right] \\
& \times J_{j+l+n}\left(\frac{a_0Mp_\perp}{(k_0\cdot p)}\right)+\Bigg[\frac{\varsigma_{-1}^{j-1}\left(E_p+M\right)}{\left(E_p+\omega-E_Q+p_z+k_z-Q_z\right)}\left(1+\frac{\lambda k_z}{\omega}\right) \\
& \times\left(\frac{a_0^2M^2\omega_0}{(k_0\cdot p)}-\frac{a_0^2M^2\omega_0}{(k_0\cdot Q)}-2\left(j+l-\tilde{l}\right)\lambda_0\omega_0\right)-\frac{\varsigma_{-1}^j\lambda a_0^2M^2\omega_0^2k_\perp p_\perp}{2\omega(k_0\cdot Q)(k_0\cdot p)}\Bigg]J_{j+l+n-1}\left(\frac{a_0Mp_\perp}{(k_0\cdot p)}\right) \\
& +\frac{\varsigma_{-1}^{j-1}\lambda_0a_0M\omega_0p_\perp}{2(k_0\cdot p)}\left(1+\frac{\lambda k_z}{\omega}\right)J_{j+l+n-2}\left(\frac{a_0Mp_\perp}{(k_0\cdot p)}\right)\Bigg]\Bigg\},
\end{aligned}
$$

(S17d)

where $W'_\Delta=\tilde{E}_p+\omega-\tilde{E}_q+\tilde{p}_z+k_z-\tilde{q}_z-2\left(j+l-\tilde{l}+\Delta\right)\lambda_0\omega_0$. The coefficients ς_Δ^δ is

$$\varsigma_\Delta^\delta=\int_{|p_\perp-k_\perp|}^{p_\perp+k_\perp}dq_\perp\frac{q_\perp^{1/2+|\Delta|}}{A_{k_\perp,p_\perp,q_\perp}}\exp\left(-\frac{4\left(q_\perp-Q_\perp\right)^2}{\tau_\perp^2}\right)J_{\tilde{l}+n+\Delta}\left(\frac{a_0m_eq_\perp}{(k_0\cdot Q)}\right)\cos\left(\delta\angle_{k_\perp p_\perp}-n\angle_{q_\perp p_\perp}\right). \quad \text{(S18)}$$

$A_{k_\perp,p_\perp,q_\perp}$ is the area of the triangle formed by $q_\perp$, $p_\perp$ and $k_\perp$. Angles $\angle_{k_\perp p_\perp}=\arccos\left(\frac{p_\perp^2+k_\perp^2-q_\perp^2}{2p_\perp k_\perp}\right)$ and $\angle_{q_\perp p_\perp}=\arccos\left(\frac{q_\perp^2+p_\perp^2-k_\perp^2}{2q_\perp p_\perp}\right)$. The AM-dependent differential scattering rate is derived from Equation (S16), $dP=\left|S_{fi}\right|^2p_\perp dp_\perp dp_zk_\perp dk_\perp dk_z$.
